# The Development of a Design and Construction Process Protocol to Support the Home Modification Process Delivered by Occupational Therapists

**DOI:** 10.1155/2018/4904379

**Published:** 2018-02-28

**Authors:** Rachel Russell, Marcus Ormerod, Rita Newton

**Affiliations:** ^1^School of Health Sciences, The University of Salford, Salford M6 6PU, UK; ^2^School of the Built Environment, The University of Salford, Salford M5 4WT, UK; ^3^School of Health Sciences, The University of Manchester, Manchester M13 9PL, UK

## Abstract

Modifying the home environments of older people as they age in place is a well-established health and social care intervention. Using design and construction methods to redress any imbalance caused by the ageing process or disability within the home environment, occupational therapists are seen as the experts in this field of practice. However, the process used by occupational therapists when modifying home environments has been criticised for being disorganised and not founded on theoretical principles and concepts underpinning the profession. To address this issue, research was conducted to develop a design and construction process protocol specifically for home modifications. A three-stage approach was taken for the analysis of qualitative data generated from an online survey, completed by 135 occupational therapists in the UK. Using both the existing occupational therapy intervention process model and the design and construction process protocol as the theoretical frameworks, a 4-phase, 9-subphase design and construction process protocol for home modifications was developed. Overall, the study is innovative in developing the first process protocol for home modifications, potentially providing occupational therapists with a systematic and effective approach to the design and delivery of home modification services for older and disabled people.

## 1. Introduction

Current government policy within the UK [1] is encouraging the design and construction industry to build new mainstream housing that supports people to successfully age in place and to reduce the architectural barriers previous design standards have caused since the majority of older and disabled people live in homes that are not designed to meet their needs [2–4]. However, current policy recognises the social and economic benefits of enabling older and disabled people to remain in their own homes by making it a statutory obligation [5, 6] for the assessment and provision of social care services to achieve this. Home modifications are one such service. Whilst home modifications can involve the removal of hazardous features, such as worn rugs, or changing the behaviour in how activities of daily living are performed [7], home modification services in the UK focus on providing “structural changes to a person's home so they can continue to live and move, or be moved, safely” (p. 410) [8]. Occupational therapists make an important contribution to the home modification process, as their professional skills in “problem solving, enablement, prevention and environmental adaptations” (p. 11) [9] are being used to help health and social care departments within local authorities deliver their legislative responsibilities for the assessment and provision of home modifications for older and disabled people.

Despite the perceived positive role of the occupational therapist in this field of practice [10] and the fact that home modifications improve the health and well-being of older people [11–13], evidence suggests that some home modifications fail to meet the client's needs [14–16] and expectations [10] and that failing to involve the client (who is usually the older person but may also be the caregiver or relative) in the decision-making process is a further cause of dissatisfaction [17, 18]. Questions have also been raised about the complexity and coordination of the home modification process because of the number of agencies and professionals involved [19–21], with the use of the analogy of a “patchwork of services,” which are relatively “unplanned and uncoordinated” in nature (p. 4) [20].

It is further suggested that people's experience of the process and satisfaction with the home modification would improve if occupational therapists had a greater understanding of their role [20, 22, 23], and the lack of available guidance and standardised assessment tools is seen as a contributing factor [16, 21, 22, 24]. This issue is further exacerbated by a lack of design and construction knowledge [8, 20, 25], leading to occupational therapists making the assumption that the modification process is simple [26]. Interestingly, evidence suggests that occupational therapists want a more standardised approach to the whole modification process [21] and that the profession should consider ways to amalgamate the occupational therapy process into the wider design and construction process [22, 23, 27]. Thus, given that occupational therapists use the principles of design and construction in interventions involving modifying the home environment in their everyday practice, the aim of this study was to develop an occupational therapy design and construction protocol for modifying home environments.

## 2. Learning from the Design and Construction Industry

Interestingly, in the 1990s, the UK design and construction industry faced similar criticisms to those discussed above, and three key factors were identified [28]. The first factor is the difficult nature of coordinating a building project requiring the careful planning, management, and coordination of a number of phases and subphases [28] and coordinating a large number of highly specialised professional groups who do not typically work alongside each other and only have a broad understanding of each other's role [29]. The second factor is the flow of information through the various sequential phases of the process [28] such that it was seen as important that each professional group understood the value of information they produced to the other professionals involved in the project and that they were aware of what information needed to flow through to the next phase and also the timing of their information such that subsequent phases were not delayed [30]. Thirdly, the involvement of end users was identified, thus ensuring that information necessary to design and construct a building to meet their needs and requirements was appropriately captured throughout the project [31, 32]. These criticisms led to the development of the generic design and construction process protocol (GDCPP) [33]. In describing the process, Cooper et al. [34] explain that the GDCPP breaks down the design and construction process into four phases, and within each phase, there are subphases; each phase and each subphase are associated with specific actions, and these actions are linked to different elements of design and construction. Each phase should be complete before moving on to the next phase. Whilst there have been no longitudinal follow-up studies investigating the long-term benefits gained from using the GDCPP, it is reported [35] that the case study sites involved in the original research continued to use the GDCPP after the formal research project was concluded.

## 3. The Need for an Occupational Therapy Design and Construction Process Protocol

When providing interventions, the College of Occupational Therapy states that “any advice or intervention provided should be based upon the most recent evidence available, best practice, or local/national guidelines and protocol” (p. 17) [36]. The occupational therapy profession has a number of generic process frameworks [37–39], and as with the design and construction industry, these processes help occupational therapists to structure the evaluation, diagnosis, treatment, and reevaluation phases of therapy. However, the occupational therapy process is generic and applied to the full range of interventions such that there is no published process which makes visible the process required for housing modifications. This should be a concern for the profession as practitioners have an ethical and professional requirement to make visible their practice such that they can demonstrate that the interventions they are providing are effective and that the person receiving the intervention is able to understand and consent to all aspects of the treatment that they are receiving [40, 41].

The assessment for, and the identification of, what home modifications are required is a complex part of occupational therapy practice, and practitioners use conceptual models as “an organising tool” to help structure and “make sense” of this process (p. 57) [42]. There is general agreement in the literature [42–44] that the Person Environment Occupation (PEO) models are the most relevant conceptual model to practitioners in this field of practice. However, there has been criticism that the traditional PEO models [45–47] do not fully capture the concepts occupational therapists require to guide effective home modification practice [48]. The Occupational Therapy Intervention Process Model [38] is used in the research reported here, and as such, these criticisms are addressed in three key ways. Firstly, the OTIPM [38] uses similar terms associated with the built environment literature such as “required space,” “required tools,” and “required actions” and similar terms used in the built environment [49] when describing the space, equipment, and objects people use to perform an activity. Secondly, unlike other PEO models [49], the OTIPM separately operationalises the process for delivering interventions. Thirdly, as with GDCPP [34], the OTIPM [38] encourages occupational therapists not to proceed to the next phase of the process until they have all the necessary information to continue, thereby reducing the risk of planning ineffective interventions.

Despite the professional [41] and ethical requirements [40] to make visible the core reasoning skills and process used within occupational therapy professional practice within the UK, there are concerns [50, 51] that very few research studies have evaluated or attempted to describe the home modification process and make visible the practice involved. Protocols have been used successfully to improve the interventions provided by occupational therapists, for example, to improve the clinical reasoning of novice practitioners using a specific assessment to identify appropriate interventions to reduce upper limb hypertonia [52]. The purpose of this study, therefore, is to develop an occupational therapy design and construction process protocol specifically for home modifications because protocols “… help clinicians focus on what is important, specify intervention procedures, delineate the theoretical rationale behind treatment, and contribute to the evolution of the intervention by explicating the reasoning process necessary to solve clinical dilemmas” (p. 712) [53].

## 4. Methodology

A survey strategy [54] was used for this study so that the home modification processes used by occupational therapists could be understood by analysing the situation in which occupational therapists undertake the process of modifying the home environment. The specific technique used to collect the survey data was an online questionnaire, as this approach provides an effective method of generating knowledge and the most efficient way of delivering the survey to a larger sample of respondents [54].

The questionnaires were designed to include both open and closed questions, capturing quantitative data about respondent attitudes and experience of the home modification process and qualitative data to capture fact-based information. Respondents were asked to consider their answers in relation to bathroom modifications as they are the most common modification [55]. A pilot study involving five experienced occupational therapists was conducted [56] to ensure the validity and reliability of the data generated, as well as ensuring that the questions could be understood by the respondents.

For the main study, purposeful sampling was chosen as an effective way to identify a sample of respondents with specific attributes necessary to generate data [57]. Inclusion criteria, alongside the rationale, are presented in Table 1. The online questionnaire was advertised through the UK College of Occupational Therapy monthly e-newsletter to all members (approximately 250 members) of the specialist section for housing. Whilst 232 questionnaires were received, only 135 met the inclusion criteria. Reasons for exclusion included the following:

1. Respondent retired from practice
2. Respondent worked outside of the UK
3. Respondent not a qualified occupational therapist
4. Respondent's main role no longer involved using home modifications as an intervention

Data analysis involved three separate stages. Firstly, a directed content analysis technique was used. Directed content analysis is a useful form of thematic analysis when validating or extending a conceptual theoretical framework, such as the occupational therapy process [58]. The OTIPM [38] acted as a theoretical framework to analyse the data. Data generated from the question “describe your role in the process of designing a bathroom modification” were downloaded into NVivo 10. Using the software, each statement from individual respondents was read and reread. Once familiar with the range of statements, the initial coding of the data involved separating the response statements into individual activities or actions performed by the respondents in their role and matching responses to one of the three phases of the OTIPM [38]. These three phases of the OTIPM [38] became the separate themes for this step of the data analysis. When using a directed content analysis, [59] states that it is important to “remember to stay grounded in the data and remain open to the possibility that, ultimately, the data and the framework may be incompatible” [59]. Therefore, codes not matched to one of the three themes were reviewed.

The second stage of the data analysis involved conceptualising the activities and actions of the respondents during the main phases of the occupational therapy process, as a home modification process. NVivo 10 software was used to produce four separate code books. Each book represented one of the themes identified from Step 1 of the directed content analysis and contained the data coded under each theme. Once familiar with the content of each book, activities and actions in each code book were matched with similar actions and activities in each of the 10 subphases of the GDCPP [33]. As with the previous stage of analysis, thematic codes not matched to the subphases were reviewed at the end of the process. The outcome of this stage of the analysis was a 4-phase, 10-subphase process used by the occupational therapist to design and construct home modification.

A third stage of the analysis was required to create an embryonic home modification process protocol framework. An iterative approach was required to generate the protocol, and a brief description of this process is given below. A framework was developed; along the top of the framework, the headings were used from the 4 phases and 10 subphases of the occupational therapy design and construction process. Running down the far left-hand side were the following principles taken from the GDCPP [33]:

- Description of the phase
- Key question
- Action needed at each phase
- Outcome of the phase

Then, using the actions and activities described by respondents in the code books generated at the second stage of the data analysis, the framework was populated. Gaps in the framework were populated by referring to *An Occupational Therapist's Guide to Home Modification Practice* [60] and the researcher's knowledge of this field of practice. To improve the trustworthiness of the data included in the framework, the principal researcher was challenged by 2 researchers not involved in this stage of the data analysis and adjustments were made accordingly.

### 4.1. Step 1 Findings

During the thematic analysis, it became evident that an additional phase not captured by the OTIPM [38] existed within the codes. This additional phase occurred between the assessment and the goal setting phase and the intervention phase. Because the respondents performed a number of actions or tasks that were not associated with the initial assessment of occupational need and the setting of goals for the intervention, nor were they related to the intervention itself. Instead, respondents performed a series of activities associated with planning the intervention; thus, the term “intervention planning phase” was developed to code these responses into a theme.

As an intervention, the home modification is not installed by the occupational therapist; however, from the responses, it was evident that a number of occupational therapy practitioners were involved in supporting the installation of the modification. Firstly, their support appeared to be essential for ensuring the health and safety of the person, for example, making the builder aware of any medical conditions which could be exacerbated by the construction methods being used to install the modification, for instance, dust exacerbating the person's respiratory condition. Secondly, some of the respondents (*n* = 13) indicated that they were involved in giving advice on the position of equipment or in purchasing specialist equipment to be installed as part of the modification. Thirdly, some respondents (*n* = 9) indicated that they had a role in providing the person with emotional support during the installation or acted as an intermediary if issues arose between the person and the builder. Therefore, using the term “intervention implementation” makes distinct that the invention is not the final installed modification alone; it involves a series of activities the occupational therapist is involved with during the phase of installing the intervention.  Table 2 presents examples of responses coded under each of the phases of the OTIPM.

### 4.2. Step 2 Findings

In Step 2 of the data analysis, NVivo 10 software was used to produce four separate code books. Each book represented one of the themes identified from Step 1 of the analysis and contained responses coded under each theme. Thematic analysis was initially attempted by looking for similarities between activities in the four main phases of the GDCPP [33]. However, it became apparent that the activities within the four main phases of the GDCPP [33] were not congruent with the activities within the four main phases of the OTIPM [38]. To overcome this issue, the activities were coded using the descriptions of the subphase of the GDCPP [33] looking for similarities in the responses in each of the four code books.

Using the abovementioned approach to the analysis, it became evident that two additional phases not captured by the GDCPP [33] existed in the responses. These two subphases occurred between subphases 1 and 2 of the GDCPP [33]. In these phases, respondents indicated a number of actions or tasks involved in analysing how the person was performing the activity in the existing environment as well as professionally reasoning what the person required in the final design. The themes “conduct an occupational performance analysis to identify the person(s) PET requirements” and “develop occupational-focused home modification goals and PET based on the person's PET requirements” were developed to capture these codes. Similarly, there were three activities described in the GDCPP [33] where no similar activity could be found in the code books; thus, no data were coded under the following themes:

- Outline feasibility
- Outline conceptual design
- Production information

The findings of this analysis are presented in Table 3 with example of responses.

To be able to compare the subphases of the GDCPP [33] and the subphases of the home modification process, the results are displayed in Table 4. The four main phases of the GDCPP [33] were differentiated by colour. By doing this, it became evident where the lack of congruence occurs between the four main phases of the GDCPP [33] and the four main phases of the home modification process. As the aim of this stage of the analysis was to conceptualise the occupational therapy practice as a design and construction process, it was necessary to resolve the issue with the lack of congruence between the four main phases so that parallels between the four main phases of the GDCPP [33] and the OTIPM [38] could be visualised, as illustrated in Table 4.

## 5. The Development of the Home Modification Process Protocol

Step 3 involved the development of a single framework based on the GDCPP [33] and the OTIPM [38]. Across the top of the framework, the 9 subphases developed from Step 2 of the analysis of the data were used to label the headings of individual columns. Populating the framework with content was an iterative process. NVivo 10 software was used to create a code book for each individual subphase of the home modification process, with each book containing the written responses coded under each of the subphases. The GDCPP Book [33] and the OTIPM Manual [38] guided the development of the content for the description of each phase, key questions needing to be asked at each subphase, and the outcome of each subphase. As such, the framework has nine subphases (0 to 8), and each of these is presented separately.

### 5.1. Subphase 0

Subphase 0, shown in Table 5, has used the GDCPP principle that a prospective client may not want to proceed with a project following an initial discussion of their need with the building professional such that the purpose of this subphase is to gather data on what has prompted the person to contact the service and whether involvement from an occupational therapist will improve the person's health and well-being.

A further principle of the GDCPP [33] is that the project manager is aware of which professionals should be involved in the process and when. Thus, taking this concept and the OTIPM [38] concept of identifying who else is involved in the person's situation, subphase 0 gathers data on who the practitioner may need to involve in later subphases of the process.

Subphase 0 has also captured the OTIPM [38] concept of making the person aware of the limitations within the practitioner's field of practice. It appeared to be important to ask this question at this phase, given the theme in the literature and the data gathered from respondents, on the influence departmental policies and resources have on the role of the practitioner.

As the GDCPP [33] is concerned with ensuring that all information is available to support the next phase of the process, the outcome subphase 0 also ensures that the practitioner has all relevant information for the next phase, in particular that the person has given consent. As consent to an assessment is an ethical and professional requirement, it appeared appropriate to include it in this phase so that when the person is first visited, they have already consented to a visit and the start of the assessment process.

### 5.2. Subphase 1

Subphase 1, shown in Table 6, captures the values the OTIPM [33] places on collaborative practice through the occupational therapy process such that the person, in collaboration with the practitioner, identifies the occupation(s) impacting upon their health and well-being.

Since the literature was critical of occupational therapists focusing on safety and function and identifying the need based on eligibility criteria, the outcome of subphase 1 assists the practitioner to identify what occupation they need to observe in the next subphase of the process. This reflects ethical practice, as the person is not arbitrarily made to perform unnecessary activities based on home-grown assessments designed to focus on safety and independence or what can or cannot be funded by the practice setting. Instead, the influence of funding arrangements is considered in subphase 4 and the feasibility study. Similarly, as the practitioner builds a collaborative relationship with the person and new data provide insights into the person's situation, subphase 1 ensures that due consideration is given to the appropriateness of the intervention in providing the person with the appropriate solution to improve their health and well-being.

### 5.3. Subphase 2

Subphase 2, shown in Table 7, has been influenced by the OTIPM [38] description of how practitioners should analyse occupational performance and participation since it is recommended that the practitioner should initially observe the person performing or participating in the occupation, identifying the strengths and weaknesses in the person's performance. Once the practitioner has these data, the OTIPM [38] describes how the practitioner can then analyse the cause of the problem based on the transaction of the person, environment, and task. This is a two-pronged approach to analysing performance and participation because it prevents the occupational therapist making assumptions about the cause of the problem. The conceptual model developed as part of the OTIPM [38] guides the type of person, environment, and occupation data the practitioner needs to collect. It should be noted that the OTIPM [38] uses the term “task” and not “occupation” in the conceptual model, thereby acknowledging that a practitioner does not objectively observe an occupation; they observe the task part of the transaction between the person and the environment. This is because only the person can experience an occupation, since it only has meaning and value to them.

### 5.4. Subphase 3

Goals are an important part of the occupational therapy process since they provide the benchmark on which the occupational therapist and person establish if the intervention has been successful. Thus, the purpose of subphase 3, shown in Table 8, is to identify those goals. Given that one of the principles of the GDCPP [33] is to collect data relevant for the success of later subphases, subphase 3 makes the distinction as to how the modification is improving health and well-being and whether it is being designed to restore, maintain, or acquire performance/participation in the person's occupation. Thus, this question prompts the practitioner to consider what impact this decision would have on the final subphase of the process.

### 5.5. Subphase 4

The purpose of subphase 4, shown in Table 9, is to conduct a feasibility study to identify how the home can be modified to improve the person's performance or participation in the occupation for which it was necessary to ensure that the protocol could accommodate a range of regional, policy, and regulatory differences between practice settings. To achieve this, the principles of the GDCPP [33] were used to develop the question of how contextual issues within the practice setting will influence the choice of design. Similarly, it was important to ensure that design decisions were made explicit to the person and documented, thus overcoming the difficulty of people not always being aware as to why certain decisions have been made.

### 5.6. Subphase 5

The development of the content from subphase 5, shown in Table 10, arose from the professional and ethical requirement of practitioners needing to ensure that the person has a full understanding of the intervention so that they are able to give informed consent to proceed with the intervention, and the questions make overt the need for the person to have a full understanding of the design before giving informed consent to proceed with the intervention.

One of the principles of the GDCPP [33] is that it provides an audit trail of the reason why decisions were made at particular subphases of the process. Thus, subphase 5 enables the occupational therapist and person to be accountable for the decisions made during the process, and it makes the information readily available if the outcomes of this subphase, or other subphases, are called into question.

### 5.7. Subphase 6

As with subphase 5, it was necessary to allow the questions to reflect the different ways modifications are funded and for the building professionals to have appropriate information to help them understand why the specific layout and requirement contained in the design plan are important in achieving the person's goals. Therefore, subphase 6, Table 11, places a duty on the occupational therapist to provide this information, thereby improving communication. Also, at subphase 6, the occupational therapist is no longer given the option to consider if a home modification approach should be taken because issues that could make a home modification inappropriate would have been identified by the person and occupational therapist earlier in the process.

### 5.8. Subphase 7

By using the principles of the GDCPP [33], subphase 7, shown in Table 12, reflects the tasks identified by respondents in the questionnaire, where their involvement was required to ensure that the person and builder were both supported during the physical construction phase of the modification.

Subphase 7 also ensures that the practitioner provides any specialist equipment that is required once the modification is installed and which could prevent the final modification from being used immediately by the person if not provided.

### 5.9. Subphase 8

Subphase 8, shown in Table 13, is an important part of the occupational therapy design and construction process. The content of subphase 8 was influenced by the requirement a number of respondents identified in ensuring that the standard of workmanship met the standards expected from the housing authority. In the GDCPP [33], the final subphase ensures that the building is handed over ensuring that the end users have an understanding of how the building operates and needs to be maintained; thus, this section ensures that the person has a similar understanding in terms of the modification. To capture concepts associated with the OTIPM process [38] and the occupational therapy process in general, the questions and outcomes of subphase 8 reflect the need to evaluate whether the goals identified in the earlier subphases have been achieved. Also, subphase 8 provides opportunity for the occupational therapist to reflect on their practice.

## 6. Discussion

As a problem-solving profession, the occupational therapy process provides the logical route that the practitioner should follow in order to provide effective interventions [61] such that practitioners are able to operationalise their professional practice [62]. From the findings of Step 1 of the data analysis, it appears that the occupational therapy process was assisting respondents to articulate their role in home modifications. For example, the quotes from R6 and R56, presented in “Findings” (although their answers differed considerably in terms of the detail provided by each respondent) still provide evidence of assessment, goal setting, and intervention phases, and in the case of R6, an evaluation phase.

The thematic analysis also raised theoretical challenges about what constitutes an intervention? The intervention has been traditionally viewed as the completed home modification [8, 63]. However, it is the skills and knowledge of the occupational therapist during all aspects of the occupational therapy process that are essential in the final design and performance of the modification, and this raises the question as to whether the occupational therapy profession should place greater emphasis on the *process* being the intervention rather than the *completed modification*. Indeed, if the process becomes the intervention, then it would be more evident as to what the intervention is and what training is required to gain the skills to carry out the intervention. By developing outcome measures that evaluate the process as the intervention, it also allows practitioners to identify which phases of the intervention were more or less effective and how the process has contributed to the person's health and well-being.

It has been possible to use the OTIPM [38] and GDCPP [33] to describe the occupational therapy process used by respondents in this area of practice. However, the outcome of this does not reflect the actual practice described by respondents, and it appears to differ in one important way, namely, the way respondents combine departmental processes with the occupational therapy process. As an example, it can be seen that respondent R29 using both phrases that are associated with the occupational therapy process (words in red) and the phrases that seem to suggest the influence of the systems, structures, and policies within the respondent's practice setting (words in blue).

*As an OT I complete an overview assessment with the service user in their home environment to identify their needs. To address these assessed needs (according to the FACS criteria), I may be required to provide adaptive equipment and in some cases recommend adaptations. If adaptations are required, I complete a referral for DFG for adaptations which, following my Manager's approval, is forwarded to the District Council & HIA or Housing Association to begin the DFG process. I provide technical diagrams and guidelines for the adaptations to ensure they can best meet the client's needs as well as completing joint site visits with technical officers if required. Once the modification is complete, I am involved with signing off the work. I am also responsible, if relevant, to obtain quotes*. (R29)

The actions of respondent R29 may not directly lead to a poorly designed modification, but previous findings [64–66] have noted how departmental policies enacted by occupational therapists have been associated with dissatisfaction with the modification. Thus, this finding raises the question as to whether practitioners are aware of how departmental structures and guidance influence their professional practice and the design options presented to the person. Again, this is an important question to answer, given the professional and ethical responsibility professionals have in ensuring that the intervention they provide has been fully explained and explored with the person, so the occupational therapist needs to be able to describe to the person how the intervention they are providing is being influenced by the practice setting.

Another important finding from the second stage of the analysis was the use of the term “assessment of need” in which respondents used their professional reasoning skills to identify occupations (activity) the person is having difficulty performing or participating in, identifying and analysing why the person is having difficulty, and analysing and identifying if a home modification will address the occupational need. From the data collected, it is not possible to establish whether in everyday practice respondents make a distinction between the different types of professional reasoning necessary to support each aspect involved in the “assessment of need” and what the consequence might be if they do not make the distinction. However, given that one principle of the GDCPP [33] is to ensure that, where possible, a subphase does not progress to the next phase until the outcome of the previous phase is achieved, the research suggests that occupational therapists are prematurely progressing through the process without all relevant data being collected and analysing as to how it might impact on the subsequent phases. If this is the case, then a process protocol for home modifications may reduce the risk of this occurring.

## 7. Conclusion

The purpose of the study was to develop a home modification process protocol by conceptualising the occupational therapy practice involved in home modifications as a design and construction process, and a number of conclusions can be drawn. Firstly, with data from the questionnaire and guided by the OTIPM [38], it was possible to both visualise and describe this process. Whilst interventions involving home modifications can be described through the occupational therapy process, it was interesting to note that practitioners have an important role in planning the design of the intervention. Furthermore, the term “intervention implementation” better describes the involvement of the occupational therapist as they are not directly responsible for the installation of the intervention themselves. Thus, the term “intervention implementation” acknowledges that installing a home modification is a dynamic process and one that the practitioner works with building professionals to achieve.

Secondly, by using the occupational therapy process for home modifications, it was then possible to use the GDCPP [33] to conceptualise the process as a home modification as four main phases based on the OTIPM [38] and 9 subphases based on the GDCPP [33]. Thirdly, using the principles of the GDCPP [33], it was possible to create a framework for the protocol, and by using an iterative process, it was possible to populate the content of this framework, which then became the home modification process protocol. This iterative process was an important part of developing the protocol because it allowed for the development of the content based on a conceptual model of practice and for issues identified in the literature to be addressed. Thus, the home modification process protocol potentially should

1. provide a systematic approach to the process of modifying the home;
2. ensure that ethical and professional practice is followed by enabling occupational therapists to verbalise and visualise their role in the process; reduce the complexity of the current process by identifying the key questions, actions, and outcome of each phase;
3. improve the effectiveness and efficiency of practice by ensuring that practitioners collect the right information, at the right time;
4. ensure that the person has choice and control through their involvement in all phases of the process;
5. guide professional reasoning based on a conceptual model of practice;
6. ensure consistency of occupational therapy practice by accommodating regional, legislative, and regulatory differences between practice settings;
7. ensure that financial constraints and other contextual issues within practice become a design consideration and not a barrier for accessing funding for a modification.

Whilst home modifiications have been a traditional area of practice for occupational therapists, the home modification process protocol is the first time this practice been described as an occupational therapy design and constuction process. Through the development of the protocol, there is the potential to address the professional [50, 51] and ethical need [40, 41] for practitioners to better understand the intervention they are providing and to be able to express their role in the design and construction of a home modification.

Importantly, this study has also raised the question as to what is the “intervention” within home modification practice? In the literature, the intervention appears to be the installed modification, and outcome measures designed to evaluate the intervention tend to be focused on how the installed modification has improved the person's performance in the occupation. However, the findings from this research have shown that each phase of the protocol is important because the outcomes from each phase can ultimately influence the final performance of, and satisfaction with, the modification. Therefore, this raises the question as to whether the home modification process is what practitioners should be defining as their intervention?

Crucially, the necessary skills and knowledge to design and construct a home modification are not taught in detail or depth at undergraduate level within occupational therapy education. Once qualified, there are training opportunities for practitioners, but these tend to be based on the knowledge and skills required to design a particular type of modification or to design a modification for a particular health condition or disability. Building the necessary knowledge of the design and construction process should therefore be reviewed within undergraduate education.

Finally, there is a need to consider how the home modification process protocol could be implemented beyond England, which was the boundary of the research reported here. Home modification is a complex area of practice, and there is a need to find ways to implement systematic assessment, intervention, and evaluation strategies within occupational therapy practice [67] The challenge for further research is that it is difficult for the process to be standardised as each country provides and funds home modifications in different ways as well as design standards and regulations also being different in each country [68].

## Figures and Tables

**Table 1:** Respondent inclusion criteria.

Inclusion criteria	Rationale for criteria
Occupational therapy	The study is interested in occupational therapy and the use of home modifications

Involved in using home modifications as an intervention	For respondents to be able to comment of the home modification process, they need to have relevant knowledge of using this as an intervention

UK-based	Different countries use different terms for describing concepts within occupational therapy, so UK knowledge was important

**Table 2:** Example of responses for the main phases of the OTIPM [38].

Main phase of the OTIPM [38]	Direct quote taken from different respondents
Assessment and goal setting	“Assessing with the person what their needs are in relation to home environment” (R2)
	“My role firstly involves an OT assessment which takes into account the goals of the individual as regards achieving the best bathroom facility for them and/or their care requirements” (R48)
	“Carry out an assessment of need, and if the assessed need results in the provision of a bathroom adaptation, would proceed to the next phase of the adaptation process” (R63)

Intervention planning	“I work with the client and technician to agree on the best possible layout to meet a person's long-term needs. This is a joint agreement with client OT, technician and builders all giving input. However, it is my role to advice on installations that may be beneficial and that the client is not aware of existing” (R3)
	“Following a functional assessment of needs, my role is to design and plan the layout and facilities in the bathroom to meet the individual's current to long-term needs” (R14)
	“Using a plan see if intended adaptation fits exploring options, i.e., shape dimensions how the client intends to use it” (R42)

Intervention implementation	“Remaining available through alterations, for site visits and answering questions as and when they arise” (R10)
	“Communicating any special needs (e.g. re dust inhalation) to surveyor/contractor” (R56)
	“Availability for consultation during the building work” (R72)

Reevaluation	“When work completed to ensure modifications are safe for client, that the work specified has been completed to a high standard and to ensure client completely happy. If not, to assist client to ensure all changes are made to ensure clients safety and ability to enjoy their new facility. Finally, there is a key role in evaluating the provision with the client and or care staff” (R6)
	“Visiting tenant once work completed to check suitability, demonstrate use of shower and other equipment and to check the adaptations meet the need” (R24)

**Table 3:** Example responses for each of the subphases of the home modification process.

Subphase	Example of responses
Demonstrate an occupational need within the person-centred performance context	“Identifying what problems exist and either what the relevant parties wish to achieve or providing information of what can be achieved (within public funding but with acknowledgement of what is available outside of public funding)” (R83)

Conceptualise the occupation need as identified by the person	“A thorough understanding of persons aspirations and their needs/wishes” (R6)

Conduct an occupational performance analysis to identify the person(s) PET requirements	“Do an initial assessment of the person and their environment looking at their functional ability and/or the needs of their carer” (R46)

Develop collaborative goal(s) and identify person, environment, and task (PET) requirements for the home modification	“Following the assessment OT recommendations discussed with the person” (R72)

Conduct substantive feasibility study for achieving the PET requirement (including funding route)	“I work with the client and technician to agree on the best possible layout to meet a person's long-term needs. This is a joint agreement with client OT, technician and builders all giving input. However, it is my role to advice on installations that may be beneficial and that the client is not aware of existing” (R3)

Obtain agreement on the full detailed design of the home modification	“Approval from service user then written options proposal, specification and CAD diagrams” (R8)

Coordinate and support procurement of the occupation-focused home modification	“Referral to District Council or RSL for DFG/minor works funding” (R100)

Construct the occupation-focused home modification	“Once work is on site, deal with any queries regarding change of layout due to unforeseen problems” (R57)

Conduct site visit to check the operation and maintenance of the occupational-focused home modification	“When work completed to ensure modifications are safe for client, that the work specified has been completed to a high standard and to ensure client completely happy. If not, to assist client to ensure all changes are made to ensure clients safety and ability to enjoy their new facility” (R6)

**Table 4:** Conceptualising the occupational therapy home modification process as a design and construction process.

Main phase of the GDCPP [33]	Subphase	Terms used in the GDCPP [33]	Activity themes generated from coding	Subphase of the home modification process	Main phase of the OTIPM [33]
Preproject	0	Demonstrating the need	Demonstrate an occupational need within the person-centred performance context	0	Evaluation
	1	Conception of need	Conceptualise the need as identified by the person	1	
	2	Outline of feasibility	Conduct an occupational performance analysis to identify the person(s) PET requirements	2	

Preproject	3	Substantive feasibility study	Develop collaborative goal(s) by identifying the detailed PET design requirement for the home modification	3	Modification planning
	4	Outline conceptual design	Conduct substantive feasibility study for achieving the PET specification (including funding route)	4	

Preconstruction	5	Full conceptual design	Obtain agreement on the full detailed design of the home modifications	5	Modification planning

Preconstruction	6	Coordinate design, procurement, and full financial authority	Coordinate and support procurement of the occupation-focused home modification	6	Modification implementation

Construction	7	Production information	Coordinate and support procurement of the occupation-focused home modification	6	Modification implementation

Construction	8	Construction	Construct the occupation-focused home modification	7	Modification implementation

After completion	9	Operation and maintenance	Conduct site visit to check the operation and maintenance of the occupational-focused home modification	8	Reevaluation

**Table 5:** Subphase 0 of home modification process protocol.

Assessment phase	Subphase 0
Description	Demonstrate an occupational need within the person-centred performance context

Key questions	What is the situation that has prompted contact with the occupational therapist/service?
	Is an occupation-focused home modification intervention appropriate for the situation?
	Is the person aware of the limitation in this practice setting?
	Should a home modification approach be taken?

Action	Identity the context of the situation
	Identify who (persons) is involved in the situation
	Identify the tasks involved in the situation
	Identify how resources and other limitations within the practice setting may affect the situation
	Identify how a collaborative relationship with the occupational therapist/service could impact on the situation

Outcomes	Referral accepted/declined
	Key referral (situational) information documented
	Person(s) aware of limitations within the OT's field of practice, that is, funding criteria for home modification
	Consent to assessment documented

**Table 6:** Subphase 1 of home modification process protocol.

Assessment phase	Subphase 1
Description	Conceptualise the occupational need as identified by the person(s)

Key questions	What is the reported occupation(s) the person(s) needs/wants to address through an occupation-focused home modification?
	Should a home modification approach be taken?

Action	Identify the specific occupation(s) the person(s) wants/needs/has to do
	Identify the person(s) occupational priorities
	Identify occupations that cannot be addressed through occupation-focused home modification intervention

Outcome	Identify the person(s) occupational priorities
	Provide advice including referral to alternative services

**Table 7:** Subphase 2 of home modification process protocol.

Assessment phase	Subphase 2
Description	Identify the person, environment, and task elements impacting on occupational performance

Key questions	How does the transaction between the person, environment, and task (PET) factors impact on occupational performance?
	Should a home modification approach be taken?

Action	Identify the actions, within the occupation(s), the person(s) does not perform effectively
	Identify actions, within the occupation(s), the person(s) does perform effectively
	Identify the elements of the person, environment, and task (PET) [38] that are affecting the person(s) occupational performance

Outcomes	Occupational performance analysis completed and effective and ineffective elements of performance documented
	PET element(s) causing effective or ineffective occupational performance documented
	PET information needed to support subphase 4 documented
	Provide advice including referral to alternative services

**Table 8:** Subphase 3 of home modification process protocol.

Intervention planning phase	Subphase 3
Description	Develop collaborative goal(s) to identify the detailed PET design requirement for the home modification

Key questions	Is the person(s) goal(s) for the modification to
	** **restore their occupational performance/participation?
	** **maintain their occupational performance/participation?
	** **develop their skills or role to perform or participate in a new occupation?
	What are the detailed PET design requirements for achieving the collaborative goals?
	Should a home modification approach be taken?

Actions	Identify, with the person(s), if the goals for the home modification are
	** **restoring their occupational performance/participation?
	** **maintaining their occupational performance/participation?
	** **developing their skills or role to perform or participate in a new occupation?
	Identify, with the person(s), how the abovementioned approach will impact on the evaluation phases
	Identify the specific “person factors/body functions” design requirements
	Identify the specific “environmental” design requirements
	Identify the specific “task” design requirements
	Identify any occupations(s) that cannot be addressed through an occupation-focused home modification

Outcomes	Person(s) has collaborated on the goals of the home modification
	Goals for home modification documented
	PET design requirements to achieve the goal(s) documented
	Reablement, rehabilitation, and/or training requirements following the completion of the home modification documented

**Table 9:** Subphase 4 of home modification process protocol.

Intervention planning phase	Subphase 4
Description	Conduct a substantive feasibility study for achieving the PET requirements (including funding route)

Key questions	What design options are there for meeting the PET requirements?
	What other factors in the person's occupational context will affect the choice of design solutions?
	Does the design proposal meet the PET requirements outlined in subphase 3?
	Should a home modification approach be taken?

Actions	Identify that the design has addressed all the requirements identified in subphase 3
	Identify that the design meets any other occupational performance context requirements
	Identify any practice setting contextual issues that will influence the person(s) choice of design solution
	Identify any potential built environment issues, in the existing space, that will impact on the PET requirements being accommodated
	Identify funding requirements for the home modification

Outcomes	Professional reasoning on the modification design solution process
	Document any issues related to the practice setting or built environment that prevent the optimum design solution being provided
	The specification related to space, space layout, and tools documented

**Table 10:** Subphase 5 of home modification process protocol.

Intervention planning phase	Subphase 5
Description	Obtain agreement on the full detailed design and specification of the home modification

Key questions	Does the full detailed design provide the solution to address the occupational performance requirements of the person?
	Do the detailed design plans and specifications provide the person with the information they need to give informed consent?
	Should a home modification approach be taken?

Actions	Ensure that the person(s) understands how the design solution addresses their occupational performance requirements
	Identify how any unmet requirements will impact on the occupational performance of the modification
	Confirm that the person(s) agrees to proceed with the design solution

Outcomes	Informed consent documented

**Table 11:** Subphase 6 of home modification process protocol.

Intervention implementation phase	Subphase 6
Description	Coordinate and support procurement of the occupation-focused home modification

Key questions	What information and action are required to procure the home modification?
	Has all the information been obtained for the builder/contractor/others to construct the home modification?

Actions	Identify and communicate information required for the procurement of the home modification
	Identity and communicate the information required for the builder/contractor/others to proceed with the construction of the home modification
	Identify and communicate what ongoing support will be required of the occupational therapist/service during construction phase

Outcomes	Funding application/support completed
	Plans, specifications, product information, and health and safety information provided to the builder and/or those involved in construction of the modification
	Agree with person and builder support being provided by the occupational therapist during construction

**Table 12:** Subphase 7 of home modification process protocol.

Intervention implementation phase	Subphase 7
Description	Construct the home modification

Key questions	Is the appropriate support being provided to the person(s) and building professional during the construction phase of the home modification?

Actions	Provide ongoing support during the construction of the home modification
	Provide and/or supply tools not part of the construction process
	Provide advice on final positioning of tools

Outcomes	Modification completed

**Table 13:** Subphase 8 of home modification process protocol.

Evaluation phase	Subphase 8
Description	Conduct site visit to check the operation and maintenance of the occupation-focused home modification

Key questions	Is the home modification operating in the way it is intended to?
	Does the home modification perform in the way that achieves the goals and requirements identified in subphase 3?
	What can we learn from the process?

Actions	Provide reablement, rehabilitation, and/or training to enable the use of the modification
	Conduct reevaluation following completion of the home modification and compare with subphase 2
	Provide training on the maintenance of the home modification
	Complete professional evaluation of the intervention and what can be learned

Outcomes	Complete and document the reablement, rehabilitation, and/or training provided
	Person(s) provided with information and documentation needed to manage the home modification
	Person(s) satisfied with the performance of the modification. Feedback documented
	Occupational therapist satisfied with the performance of the modification completed. Outcome documented
	Modification resolves the occupational need identified in subphase 3. Case closed
